# Molecular breast cancer subtypes prevalence in an indigenous Sub Saharan African population

**DOI:** 10.11604/pamj.2014.17.249.330

**Published:** 2014-04-05

**Authors:** Moses Galukande, Henry Wabinga, Florence Mirembe, Charles Karamagi, Alexzander Asea

**Affiliations:** 1 Department of Surgery, College of Health Sciences, Makerere University, Kampala, Uganda; 2 Department of Pathology, College of Health Sciences, Makerere University, Kampala, Uganda; 3 Department of Obstetrics and Gynecology, College of Health Sciences, Makerere University, Kampala, Uganda; 4 Clinical Epidemiology Unit, College of Health Sciences, Makerere University, Kampala, Uganda; 5 The Texas A & M Health Science Center College of Medicine, USA

**Keywords:** Molecular subtypes, breast Cancer, triple negative breast cancer

## Abstract

**Introduction:**

Sub-Saharan Africa is predicted to face an unprecedented growth of cancers including breast cancer. There are indications of a significant burden of aggressive and late stage breast disease among premenopausal women in sub-Saharan Africa; because hormonal status tests are not routinely done, many women are given anti-hormonal therapy empirically. There is paucity of data on breast cancer molecular subtypes and their characteristics among women in sub Saharan Africa. The objective is to determine the prevalence of breast cancer molecular phenotypes among Ugandan women.

**Methods:**

This was a cross sectional descriptive study, conducted at a tertiary hospital in Africa. Eligible participants’ formalin fixed and paraffin embedded sections were evaluated. H & E stains and Immunochemistry (Estrogen Receptor (ER), Progesterone Receptor (PR), Human Epidermal growth factor Receptor (HER2)) were performed. Ethical approval was obtained.

**Results:**

A total of 226 patient samples were evaluated. The mean age was 45 years (SD 14);the prevalence of Triple Negative Breast Cancer (TNBC) was 34% (77/226), Luminal A 38% (83/226), HER2 positive was 22% (49/226), and Luminal B was 5% (13/226). High-grade (III) tumors were 68%, stage III and IV constituted 75% of presentations. Histological type was mostly invasive ductal carcinoma. Most patients (55%) were from rural areas.

**Conclusion:**

Ugandan women had an over representation of TNBC and high-grade breast tumors. Underlying reasons ought to be investigated. The empirical use of tamoxifen (anti-hormonal therapy) should be reexamined.

## Introduction

Women in sub-Saharan Africa are experiencing a rapidly increasing burden of aggressive breast cancer [1–6]. In fact there is evidence suggesting an emerging epidemic of breast cancer in Africa [7, 8]. There is some evidence to suggest over re-presentation of unfavorable breast cancer phenotypes occurring mostly among pre-menopausal women in sub-Saharan Africa [2, 4, 9, 10]. Although the incidence of breast cancer is lower in developing than is in developed countries, in Uganda breast cancer incidence has nearly tripled in the past three decades [6] and there are more women with breast cancer below the age of 50 years. This is a much lower age compared to the western nations [6, 9, 11]. Similarly, in a recent British study black women presented at a younger age with a higher frequency of grade III and ER negative tumors. In addition there are poorer outcomes among black women than there are among white women with breast cancer [12]. Although, there are some data to explain these disparities [7–9, 11]. The reasons for the differences are not yet fully established, therefore more data are needed[13, 14]. Gene expression profiling tools and studies have identified breast cancer sub types and demonstrated the ability to predict clinical outcomes independent of other prognostic factors. These studies have led to the classification of breast carcinoma as luminal A, luminal B, human epidermal group factor receptor 2 (Her 2) - over expression and triple negative. These differ markedly in prognosis [12, 15–17]. Routine classification of breast cancer (based on hormonal receptor status) is practiced in most developed countries. In countries where severe resource constraints exist, the practice of profiling breast cancer by hormonal receptor status is not routinely done and therefore this characterization of breast cancers by molecular subtypes is not well documented. In the absence of hormonal status tests nearly all patients receive anti-hormonal therapy (tamoxifen) routinely. In Uganda the proportions of these molecular breast cancer subtypes are largely unknown. The aim of this study therefore was to determine the prevalence of molecular sub types among Ugandan women diagnosed with breast cancer.

Country Overview: Uganda is a land-locked country straddling the equator in eastern Africa. The country is 241,040 Km^2^ and currently has a population of over 32 million people [18]. Uganda is due to double its population (starting with 2,006 numbers) by 2037[19]. Almost one-third of the country lives in poverty (defined as living on less than US $1.25/day) [18]. A total of 85% of the population live in rural areas, and most of them work in the agriculture sector [20]. Uganda ranks at 143 among the 169 countries surveyed for the 2010 Human Development Index [21, 22]. The Ugandan Health system is developed with public and private providers, most health care is free and referral for cancer care is mostly to Mulago National referral and teaching hospital for the College of Health Sciences. The Makerere Pathology department is the only public unit providing free histopathology services in the country.

## Methods

**Design:** Across sectional descriptive study; data were collected for the periods 2004-2007 and 2011- 2012.

**Setting:** The study was conducted at two institutions; Mulago hospital, the national referral hospital in Uganda with a 1500 bed capacity and with over 400 physicians and the Uganda Cancer Institute (UCI) located on the same campus as Mulago hospital. UCI is the only specialized public cancer treatment center in Uganda. Over the study period it was estimated that close to 500 patients could have presented to both institutions with invasive breast cancer.

**Study population and Sampling procedure:** Women with a histologically confirmed diagnosis of invasive breast cancer over the study period were included in the study. Those with insufficient clinical information were excluded from the analysis. The participants recruited prospectively had their samples obtained as core biopsies following the hospital standard protocol. The core biopsies were taken after infiltration with 2-3cc of 2% lignocaine but without US scans guidance. A BARD^®^ magnum^®^ device with 16G or 18G needles was used to take the core biopsies. Three cores were taken from each patient along with accompanying clinical details. All biopsy samples were fixed in 10% freshly prepared formalin. The fixed tissue samples were sent to the laboratory for histological analysis and immunochemistry. In addition, samples were retrieved from the specimen archives. Tumor sections were cut from the tissue blocks and a slide stained with haemotoxylin-eosin (H & E). A pathologist to confirm presence of cancer and histological grade reviewed each subject. Case notes were reviewed for additional information including age, residency (rural or urban) and stage. Slides were also stained for ER, PR and HER2/neu immediately after sectioning.

**Evaluation of ER, PR & HER2/neu status and Quality Assurance:** TNBC was defined as all ER, PR and HER2 receptors staining negative. HER2 positives were ER & PR negative and with HER2 positive staining. Luminal A was either ER or PR positive with a negative HER2. Luminal B was either ER positive /negative and or PR positive with a positive HER2. We used the quality assurance guidelines of the College of American Pathologists (CAP). The laboratory control tissue had a proven positive slide for each of the antibodies. A section of the positive and negative controls were used at every run of the day. Paraffin block specimens were cut into four sections and mounted on positively charged slides. The slides were paraffinized and rehydrated in xylene followed by graded alcohols, then washed in Tris buffered saline. The immunochemistry assays were performed using an immunostainer with antibodies and antigen unmasking. Appropriate negative controls for the immuno-staining were prepared by omitting the primary antibody step. The results were scored semi quantitatively using Reiner's four point scale based on intensity and percentage of IHC reaction [23], HER2 staining were evaluated according to manufacturer's instructions. Antibodies used were: ER (clone SP-I), ASR PR (clone Y85), ASR and HER2/neu (c-erbB-2), clone CB-11). (Cell marquee corporation, Rocklin, CA) ER/PR scoring system staining of <5% of tumor cell nuclei was considered negative. Both border line and overtly positive stains were considered positive. Her2/neu; negative was considered when no staining was observed or membrane staining was <10% of the tumor cells.

**Evaluation of histopathological features:** H & E staining was performed first to confirm diagnosis of invasive breast cancer before immunostaining. The histological type and grade were determined. An experienced consultant pathologist reviewed all the histological slides and the tumors were classified according to Nottingham modification of the Scorff Bloom Richardson criteria [24]. Based on histology, tumors were classified into the following groups: invasive ductal carcinoma (NoS), lobular, medullary, papillary, and colloid.

**Data entry and analysis:** A database was developed in SPSS version 17 for entry and analysis. Data on clinicopathological features among the two age groups >50 and

**Ethical considerations:** The study was approved by the Makerere College of Health Sciences, School of Medicine Research and Ethics Committee and was duly registered with Uganda National Council of Science and Technology. All patients in the prospective arm gave written informed consent. The Ethics committee waived written consent for the retrospective arm. The juvenile in the study had consent given on her behalf by the parent.

## Results

**Figure 1:**
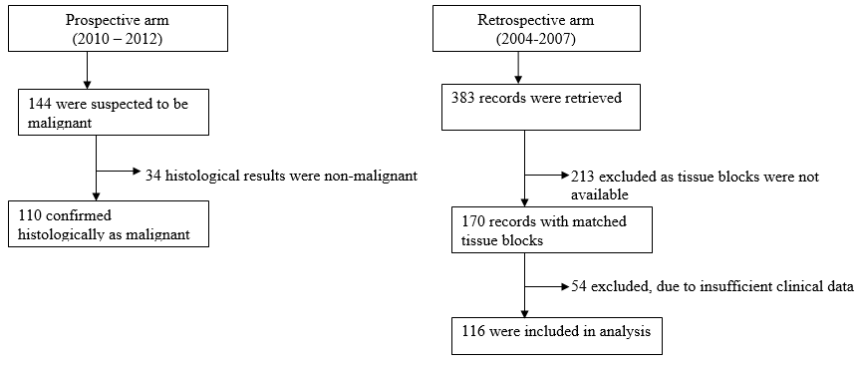
Breast Cancer patients included in analysis

A total of 226 cases were analysed. Out of the 144 patients screened for malignant disease, for the period 2010-2012, 34 posted non malignant histology results and were not included in the analysis. Of the estimated 383 breast cases that presented at both institutions during the 2004 -2007 period, 170 samples of confirmed breast cancer diagnosis (formalin fixed paraffin embedded tumor blocks) were available, 54 were excluded due to insufficient clinical data. In total 116 were included in the data analysis (Figure 1).

The mean age at presentation was 45 years (SD 14). TNBC constituted 34% of all cases (77/226), HER2+22% (49/226), Luminal A 38% (83/226) and Luminal B 5% (13/226). The majority of the women were premenopausal 152/226 (67%), considering 50year as the proxy for menopause. Women presented with stages III and IV disease were (73-88%). The predominant histological type was invasive ductal carcinoma (NoS) 93 -96% 2. One patient was a juvenile (13years old), the rest were 27 years and above (Table 1, Table 2).

**Table 1:** Clinical and Biological tumor characteristics for the 226 study participants

Characteristics	Number (%)
Age	45 SD 14
**Age**	
≤ 50 years	152
> 50 years	69
**Tumor stage**	
I	4
II	24
III	110
IV	31
Missing	57
**Histology**	
Invasive ductal carcinoma	198
Lobular	14
Others	12
Missing	2
**Tumor grade**	
I	16
II	57
III	149
Missing	4
**Tumor stage by setting**	
**Rural**	
I	0
II	12
III	40
IV	7
**Urban**	
I	4
II	9
III	36
IV	3

**Table 2:** Patient characteristics by Molecular sub types Uganda Breast Cancer study 2013

Characteristic	Over all N=226	TNBC N=77	HER2+N=49	Luminal A N=83	Luminal B N=13
**Age characteristics**					
Mean age (years)	45 (SD 14)				
Women ≤ 50years†	152	38	24	32	11
Women > 50years††	69	31	18	15	1
Location*					
Rural	105	48	22	29	6
Urban	74	26	17	28	5
**Tumor stage^**					
I	4	1	4	3	0
II	24	11	4	8	1
III	110	24	22	5	7
IV	31	14	6	9	2
Tumor grade **					
I	16	10	1	4	0
II	57	26	5	19	7
III	149	41	43	60	6

† 47missing

* 45 missing

^ 57 missing

** 4 missing

†† Missing

## Discussion

We set out to determine the prevalence of molecular sub types of breast cancer among Ugandan women presenting at the national referral hospital and Uganda Cancer Institute. We found a 2 to 3 fold over representation of TNBC (34%) when compared to data from western countries where the prevalence of TNBC is 12-17% or less [25–27]. The histological grades were mostly grade III; the clinical stages at presentation were mostly III and IV (advanced disease). Her2 positive and TNBC tumors together constituted close to 60% of the breast cancer molecular sub types. The majority of women were 50years and below. Patients with HER2+ exhibit poor survival rates, more so in the absence of targeted therapies, which is the case in most resource poor settings.

Our study findings add to the existing evidence that breast cancer in sub-Saharan Africa is characterized by aggressive disease, advanced stage at presentation and with a significant proportion among young women under the age of 50 years [1, 4, 5, 7, 28–31]. There were no significant differences in the disease stage between the rural and the urban study participants, even though it is anticipated that the rural patients face significant travel and distance barriers to get to the diagnostic and treatment centers. HER2 and TNBC subtype represent the more unfavorable subtypes in as far as treatment outcomes are concerned. TNBC exhibits characteristics, which are quite distinct from other forms of breast cancer. TNBC presents as an aggressive disease, recurring and metastasizing more often than other kinds of breast cancers [27]. The occurrence of different prevalence rates of subtypes may not only suggest heterogeneity in oncogenesis, but also it part explains the differences in survival outcomes observed between the races (black Africans and Caucasians). Whereas, survival outcomes in resource rich countries are improved by new molecular target therapies such as Trastuzumab and lapatinib used in HER2+tumors, these therapies are hardly accessible by women in poor countries. Consistent with other studies we found that the ER negative breast cancer subtypes, which include TNBC and some of the HER2+, were associated with higher grade and higher proliferative capacity [32, 33]. The reasons for the relatively high TNBC and HER2 proportions are not yet fully understood. Some studies suggest that these disparities may be explained by genetic differences such as unidentified founder mutations in breast cancer [34–36]. Studies to explore gene mutation patterns may reveal the much needed clues [32, 37, 38]. The mean age in this study was 45 years at diagnosis, 10-15years earlier than in breast cancer populations in western industrialized countries [11, 12, 39]. The youngest participant was 13 years old. This 13-year old was an outlier, the next youngest woman with breast cancer was a 27-year old. The majority of patients were premenopausal taking 50years as a proxy for menopause [40, 41], the overwhelmingly large premenopausal proportions can be explained in part by the fact that the Ugandan population is largely young as described earlier in the study context and Uganda's life expectancy is at 53 years.

HER2 protein over expression is seen in 10% to 35% of human breast cancers and is associated with amplification of the gene in more than 96% of cases [25, 26, 28, 29]. In this study the representation was 22%. Her2 over expression might be associated with reduced or even adverse response to anti-hormonal therapy. In addition to being a prognostic indicator Her2 over expression and amplification are important in determining treatment strategies, survival and response to treatment. The combined prevalence of TNBC and HER2+was close to 60%, it therefore follows that the routine (systematic) use of tamoxifen (anti hormonal therapy) without prior receptor status testing as it happens in most resource limited sub-Saharan countries [42], may not be beneficial to the majority of women under treatment. To many it is a source of false hope and a waste of meager resources. Endocrine therapy using tamoxifen, a selective ER modulator and aromatase inhibitors, which ablate peripheral estrogen synthesis, has been shown to substantially improve disease free survival. We now know that initial or acquired resistance to endocrine therapies may occur with tumors recurring as metastatic. The tumors in this study were mostly stages III & IV. Even though we didn't investigate endocrine resistance it is possible that there could be some endocrine resistance that developed as the disease progressed (to stage IV) [43, 44]. These findings therefore call for a wider scale of data exploration perhaps from multiple sites and a rethink of how breast cancer is currently being managed in sub Saharan Africa.

### Study limitations

Previously TNBC and basal like breast cancers were effectively synonymous [32, 33], we now know that clinical micro assay and immuno-histochemical data show that this is not the case [25]. In this study we did not differentiate TNBC and basal like breast cancer because the majority of basal like cancers are also triple negative and the majority of TNBC (80%) also basal like [45]. We are aware that the high frequency of hormone receptor negativity in the retrospective arm may have been influenced to some extent by antigen degradation of archival materials [3, 8, 46]. To mitigate that influence high standards of laboratory testing processes were followed and the specimen storage period was short (less than 10 years). Hospital based studies may not be representative of the entire population, though in this case there was a fair representation of participants from all the four major regions of the country, giving a high level of confidence about the generalizability of these findings.

## Conclusion

There was an over representation of unfavorable molecular sub types for breast cancer among this indigenous group of East African (mostly premenopausal) women. The empirical use of anti-hormonal therapy without prior receptor status testing should be reexamined. Further and larger studies are urgently required to elucidate the oncogenesis breast cancer sub types among black sub-Saharan African women.
